# The Internal Dynamics of Fibrinogen and Its Implications for Coagulation and Adsorption

**DOI:** 10.1371/journal.pcbi.1004346

**Published:** 2015-09-14

**Authors:** Stephan Köhler, Friederike Schmid, Giovanni Settanni

**Affiliations:** 1 Institut für Physik, Johannes Gutenberg–Universität Mainz, Mainz, Germany; 2 Graduate School Materials Science in Mainz, Mainz, Germany; 3 Max Planck Graduate Center mit der Johannes Gutenberg-Universität Mainz, Mainz, Germany; UC San Diego, UNITED STATES

## Abstract

Fibrinogen is a serum multi-chain protein which, when activated, aggregates to form fibrin, one of the main components of a blood clot. Fibrinolysis controls blood clot dissolution through the action of the enzyme plasmin, which cleaves fibrin at specific locations. Although the main biochemical factors involved in fibrin formation and lysis have been identified, a clear mechanistic picture of how these processes take place is not available yet. This picture would be instrumental, for example, for the design of improved thrombolytic or anti-haemorrhagic strategies, as well as, materials with improved biocompatibility. Here, we present extensive molecular dynamics simulations of fibrinogen which reveal large bending motions centered at a hinge point in the coiled-coil regions of the molecule. This feature, likely conserved across vertebrates according to our analysis, suggests an explanation for the mechanism of exposure to lysis of the plasmin cleavage sites on fibrinogen coiled-coil region. It also explains the conformational variability of fibrinogen observed during its adsorption on inorganic surfaces and it is supposed to play a major role in the determination of the hydrodynamic properties of fibrinogen. In addition the simulations suggest how the dynamics of the D region of fibrinogen may contribute to the allosteric regulation of the blood coagulation cascade through a dynamic coupling between the a- and b-holes, important for fibrin polymerization, and the integrin binding site P1.

## Author Summary

Fibrinogen, a protein found in the blood of vertebrates, when activated, aggregates and forms fibrin fibers, the basis of a blood clot. Clots are broken down by the enzyme plasmin, which cuts fibrin fibers at specific places, thus helping the regulation of clot persistence. A mechanistic understanding of fibrin degradation by plasmin is still missing. An important determinant of this process might be the flexibility of fibrinogen. The flexible nature of fibrinogen is reported, for example, by the great variety of conformations observed when fibrinogen adsorbs on material surfaces. However, limits in the spatial resolution of these experiments preclude the identification of the atomistic mechanism behind this flexibility. Here, we perform computer simulations that help identifying with atomistic detail large bending motions occurring at a specific hinge on the molecule. We show how these bending motions can explain the variable conformations observed in experiments and how they help exposing sites where plasmin can cut fibrinogen. Furthermore, our simulations let us identify cooperative effects involving several distant parts of fibrinogen that may play a role in the assembly of fibrin fibers. Both the bending and the cooperative effects, thus, represent potential mechanisms for the regulation of blood clotting.

## Introduction

Fibrinogen (Fg) is a 340kD multi-chain glyco-protein which can polymerize into fibrin, one of the main components of blood clots. Fibrin formation and lysis (fibrinolysis) are tightly controlled processes along the pathway leading to coagulation [1]. Fg, once activated by thrombin, which cleaves the fibrinopeptide A and B (FpA, FpB), exposes specific A- and B-knobs which bind to the corresponding a- and b-holes of neighbor Fg molecules and initiate the fibrin polymerization process. Fibrin is later stabilized by additional non-covalent and covalent interactions. By further interacting with other blood components through its integrin binding sites, fibrin plays an important role in regulating coagulation and immune response. Fibrinolysis on the other hand is effected by plasmin, which cleaves fibrin on specific cleavage points in a well defined temporal sequence [2–4].

**Fig 1:**
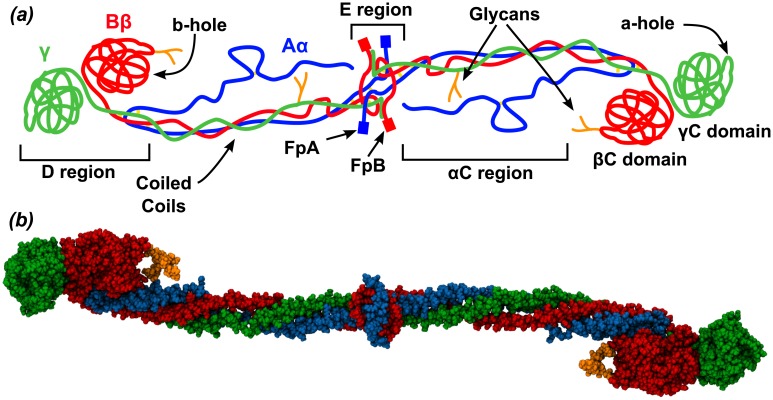
The fibrinogen molecule. *(a)* Schematic representation of the fibrinogen molecule. The three chains of Fg, A*α*, B*β* and *γ* are shown in blue, red and green, respectively. *(b)* Van der Waals representation of the crystallographic structure (pdb 3GHG) of Fg, color coded as in *(a)*. Carbohydrates are in orange. The *α*C region and the FpA and FpB peptides were not resolved in the crystal structure.

The elongated structure of human Fg, as shown by the crystallographic data [5], is formed by two symmetric units which dimerize through a central globular E region. Each symmetric unit (protomer) is constituted by 3 peptide chains A*α*, B*β* and *γ* which depart from their N-terminal region (E region), form an elongated coiled-coil region, and end into two globular domains forming the D region (Fig 1). The C terminal segment of the A*α* chain, i.e. the *α*C region, as well as the N-terminal parts of chain A*α* and B*β*, including FpA and FpB, are mostly disordered (thus, not resolved in the crystal).

Although the available crystallographic structures of Fg show a relatively limited variability, atomic force microscopy images of adsorbed Fg on several surfaces reveal a large degree of conformational flexibility. Indeed, the typical tri-nodular structure of Fg, as observed in adsorption studies, where the three nodules correspond to the two D regions and the central E region, is very variable [6], and the angle formed by the three nodules has a wide distribution [7, 8]. The source of this conformational flexibility at the molecular level is not well understood. Early sequence analysis [9] and comparison of several crystallographic structures of Fg [5, 10, 11] suggested the presence of a hinge point in the middle of the coiled-coil regions connecting the E and D regions. However, the role of this hinge point and the extent of flexibility that it confers to the Fg molecule has not yet been described.

Fg is one of the most abundant serum proteins initially adsorbing on foreign surfaces in contact with blood [12, 13], and it plays a crucial role in determining the inflammatory response to the material [14, 15]. In the case of nanoparticles, which have been the subject of intense research for their use in nanomedicine [16, 17], Fg contributes with other serum proteins, upon pre-incubation in blood, to form a protein corona surrounding the particle and determining its fate in its clinical use, i.e., circulation halftime, cell uptake, etc. [18]. Simplified models of Fg have been developed based on its hydrodynamic [19] and adsorption properties [20, 21] and used to study adsorption on solid surfaces. Similar models have been used to study the competitive adsorption of serum proteins on material surfaces and, in particular, on nanoparticles [22]. In these models, as well as in models of fibrin polymerization [23], the internal flexibility of Fg is either ignored or treated approximately, although it may play a very important role especially in the characterization of its hydrodynamic properties.

Fg, in the polymerised form of fibrin, is a structure subjected to mechanical tension. For this reason early simulation work on Fg focussed on its mechanical properties under external stress [24–27]. Here, instead, we report the results of extensive molecular dynamics (MD) simulations performed on Fg in solution. The simulations allow for the identification of large bending motions centered at a hinge point on the coiled-coil region of Fg. We also present an extensive sequence analysis of Fg across vertebrate organisms which suggests that the bending motions associated with the hinge play one or more functional roles. The simulations indicate that one of these roles may consist in the exposure of plasmin cleavage sites on the coiled-coil region. From the simulation results we construct a simplified representation of the internal flexibility of Fg and use it to fit and explain experimental data on conformational distribution of the molecule adsorbed on mica. The results of the fit point to an asymmetry in the adsorption properties of the different sides of Fg, which can be explained by the presence of large charged patches that are unevenly distributed on the surface of the globular domains of the molecule. In addition, the simulation data allow us to characterize the dynamical properties of the D region of Fg involved in fibrin formation and immune response, highlighting the presence of coupled motions between the a- and b-holes and the integrin P1 binding site.

## Results and Discussion

We have performed several atomistic molecular dynamics simulations of Fg, either in its full dimeric state or considering only one of the two symmetric protomers. In either case we have simulated glycosilated and unglycosilated constructs. Each construct has been immersed in a periodic box of water molecules at physiological ion concentration (see Methods section for details). The cumulative time length of the simulations reaches 1.3 *μ*s with several continuous stretches of simulation reaching 0.2 *μ*s.

### Hinge in the coiled-coil region

**Fig 2:**
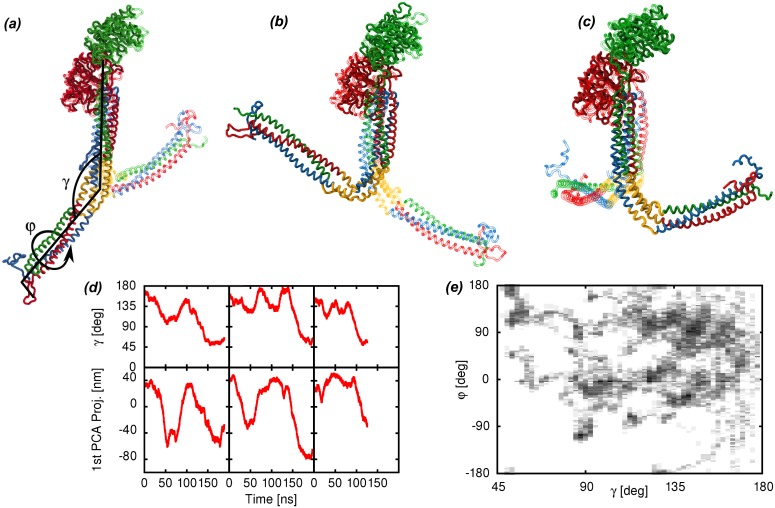
Characterization of the large bending motions of fibrinogen. *(a)*–*(c)* Dominant PCA modes of the Fg protomer with the hinge region highlighted in yellow (chains colored according to Fig 1). For each PCA mode, the two structures with the largest (solid) and smallest (transparent) projection along the PCA mode are represented. An illustration of the bending angle *γ* and the torsion angle *φ* is superimposed to the first PCA mode. The three groups of atoms used to define the *γ* angle are the E region (*α*50–58, *β*82–90, *γ*23–31), the hinge region (*α*99–110, *β*130–155, *γ*70–100) and the D region (*β*200–458, *γ*140–394). The four groups of atoms used to define the *φ* are one part of the E region (*α*50–58, *γ*23–31), another part of the E region (*β*82–90, *γ*23–31), the hinge region (*α*99–110, *β*130–155, *γ*70–100) and the D region (*β*200–458, *γ*140–394)*(d)* Time series of the *γ* angle and of the projection of the trajectories along the first PCA component from selected simulation runs. The plots show both the reversibility of the motion and the time scale along which it occurs. *(e)* Distribution of the bending angle *γ* and dihedral angle *φ* around the hinge of the Fg protomer as observed in the present simulations. The elongated conformation of Fg observed in the crystals correspond to a *γ* angle close to 160°.

Fg undergoes large bending motions in all the simulations that we have performed. Principal component analysis (PCA) is used to quantify these motions. The dominant principal components of motions (PCA modes, Fig 2*(a)*–2*(c)*) of the Fg protomer are the same in all sampled trajectories as revealed by a large overlap (see Methods section) between the three dominant modes ranging from 0.6 to 0.9 between simulation subsets. In particular, the large overlap observed between the dominant PCA modes in both mono- and di-glycosilated and unglycosilated Fg protomer trajectories shows that the carbohydrate clusters do not affect the large scale dynamics of Fg in solution. Similarly, the dimerization state does not induce any noticeable change in the large scale motion of Fg. Dimer and monomer simulations show consistent hinge bending and the PCA-mode overlap between isolated protomer and dimerised protomer simulations is large. The difference between the dimer and protomer simulations is limited to the dynamics of the dimerization interface, where the absence of the disulphide bridges with the other protomer results in expectedly larger root mean square fluctuations (RMSF) localized to the residues *α*27–44 *β* 58–75 and *γ* 14–18 (see S1 Fig in Supplementary Information). Because of the overlap of the largest PCA modes in the different simulation sets, the analysis presented here is done using all the available data merged together in a single set, which improves the statistical significance of the results. The first three PCA modes span the degrees of freedom associated with bending at a hinge point in the coiled-coil region (Fig 2*(a)*–2*(c)*), while the 4th PCA mode is related to a pure torsion of the coiled coil along its axis (not shown). The motions are reversible as shown by the time series of the PCA projections (Fig 2*(d)*) Lower ranking PCA modes provide smaller contributions to the overall variance so they will not be analyzed further.

The program DynDom [28], applied to the extremal structures observed along the first PCA mode (Fig 2*(a)*) of the Fg protomer, has been used to identify the regions of the molecule which are more rigid in our simulations, as well as the connecting hinge regions. DynDom reports the presence of two relatively rigid regions, separated by a hinge. The E region and the N-terminal part of the coiled-coil region represent one of the two rigid domains, while the C-terminal part of the coiled-coil region along with the D region represent the second. The hinge region is located approximately in the middle of the coiled-coil region and includes the break in the *α*-helical structure of the *γ* chain, which gives rise to a flexible loop (residues *γ*70–78), along with the neighbor residues on the A*α* and B*β* chains (Fig 2*(a)*–2*(c)*). The break in the *α*-helical structure of the *γ*-chain is facilitated by two proline residues.

The bending around the identified hinge can be described by a bending angle *γ* and a torsion angle *φ* defined using groups of atoms from the E region, the hinge region and the D region (Fig 2*(a)*). The *γ* and *φ* angles strongly correlate with the projections along the dominant PCA modes: *γ* shows a linear correlation coefficient of 0.96 with a linear combination of the first 3 PCA modes (1st-2nd-3rd/10, see Methods section), while *φ* has a 0.73 linear correlation coefficient with a linear combination of the 1st, 2nd and 4th PCA modes (1st/3+2nd/2+4th/2). Our simulation data show a consistent and significant bending occurring at the hinge region and reaching bending angles below 90 degrees (Fig 2*(e)*). The time it takes for the Fg structure to reach a bending angle below 110 deg from conformations similar to the crystal structure (bending angle above 150 deg) is 19±1 ns along the trajectories, averaged over the 12 observed events (see Fig 2*(d)* and S1 Movie in Supplementary Information, for examples). The reverse process occurs twice in the simulations, taking 20 and 26 ns. The simulations of the full Fg dimer do not show significant correlations between the angle values observed at the two hinges.

**Fig 3:**
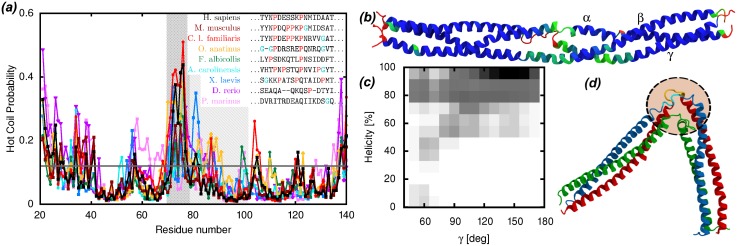
Functional role of the bending motions in the coiled-coil region of fibrinogen. *(a)* DisEMBL “hot coil” predictions for the sequences of the *γ* chain from several vertebrates, highlighting the fact that the flexibility of the non-helical loop is a conserved feature. The hinge region is shaded and, within that region, the non-helical loop segment is dark shaded. The inset legend reports the sequence alignment of the non-helical loop region across the same vertebrates, highlighting the content of glycine and proline residues. *(b)* Cartoon representation of the coiled-coil region of Fg colored according to the fraction of the simulation time spent in an *α*-helical conformation (red = 0, green = 0.85, blue = 1). The N-termini of the segments are on the left. The regions with lower helical fraction are in good agreement with regions with lower protection factors as determined in H/D exchange experiments [29]. *(c)* Probability distribution of the fraction of helical residues around the A*α*104–105 and B*β*133–134 plasmin cleavage sites as a function of the bending angle *γ*. Dark shades correspond to high probability. The three residues preceding and following the cleavage sites (i.e., A*α*102–107 and B*β*131–136) have been included in the calculation of the helicity. Larger bending (lower *γ* angle) correlates with lower helical content. *(d)* Snapshot of the conformation of the bent coiled-coil region (chains colored as in Fig 1) showing the disrupted secondary structure around the plasmin cleavage sites (rendered yellow and cyan inside the dashed circle).

Comparison of the crystallographic structures of Fg coiled-coil regions from various organisms already hinted at the presence of a flexible hinge [5]. This hypothesis is also supported by hydrogen-deuterium exchange experiments [29]. The latter are in good agreement with our simulations: amino acids from the coiled-coil region with lower helical probability in the simulations (Fig 3*(b)*) correspond to amino acids with low protection factors in the experiments. Our simulations help to clarify the fact that the presence of a flexible hinge is an intrinsic feature of the coiled-coil region, and not an artefact due to sequence or crystal-packing differences in the compared crystal structures. The hinge is positioned on the non-helical segment of the *γ* chain (*γ*70–78), most probably due to the resulting reduction in the stiffness of the coiled coil. This segment is non-helical also in the other crystallized Fg structures [10, 11]. In addition, this segment has markedly helix-breaking features in most of the available Fg sequences from vertebrates that we have analyzed, showing a large density of proline and glycine residues (Fig 3*(a)*).

As can be seen in Fig 3*(a)*, the appearance of prolines characteristic for the hinge region happens in three stages during evolution. The lamprey (*Petromyzon marinus*) has no proline, fish (*Danio rerio*) have a single proline and tetrapods have two or more. These steps coincide with major changes in the clotting cascade, namely the appearance of the intrinsic pathway in jawed vertebrates and the addition of the contact pathway (linking blood clotting and immune response) in tetrapods [30–32]. We used the program DisEMBL [33] to analyze the Fg sequences and identify disordered or loop segments in the coiled-coil region. The region corresponding to the non-helical segment of the *γ* chain is marked as a hot-loop with high probability in most of the sequences, with the exception of lamprey Fg which, as mentioned above, is known to have a simpler coagulation mechanisms than the other vertebrates [31] (Fig 3*(a)*). This analysis supports the idea that the non-helical segment of the *γ* chain provides a function that is strongly conserved across vertebrates. Our simulations suggest that this conserved function is linked to the bending motion of the coiled-coil region.

Besides providing flexibility to the individual Fg molecules as well as the fibrin fibers [5], the bending at the hinge may help expose the plasmin cleavage sites located nearby on the coiled-coil region, an hypothesis already proposed in ref. [9]. Our simulations strongly support this hypothesis showing that the *α*-helical structure around the plasmin cleavage sites A*α*104–105 and B*β*133–134 is partly disrupted by the bending motions, and the exposure to the solvent of the involved peptide bonds increases (Fig 3*(c)* and 3*(d)*). The *α*-helical structure lost during bending is generally replaced by coil structure in our simulations and not by extended *β*-sheet structure as observed in experiments [34] and simulations [35] of fibrin subject to tension. The observation of the transition to extended *β*-sheet may then be linked to the presence of tension along the molecule and/or require significantly larger times than those simulated here to occur spontaneously. It is known that the fibrin molecules straighten when they are integrated into protofibrils [36]. This does not mean that the flexibility of individual molecules is lost. The twisting of fibrin fibers [37] compresses molecules in the center of the fiber and stretches them on the perimeter. If the hinge bending is necessary to accommodate such deformation, it is reasonable to believe that the bending motions at the hinge may actually be reduced by tension applied along the fiber axis. Thus, fibrinolysis assisted by the bending motions at the hinge may selectively take place on fibrin molecules subject to reduced tension. This hypothesis is supported by experimental evidence indicating reduced plasmin fibrinolytic effectiveness on fibrin fibers subject to mechanical tension [38].

Bending may also play an important role in fibrin polymerization. According to the recently proposed Y-ladder model of fibrin polymerization [23], in the early stages of the process, the protofibril grows as a single strand where each monomer is connected to the next by a single A-knob-a-hole bond between the E domain of one molecule and the D domain of the next in the chain, giving rise to a Y-ladder structure. The latter is later transformed in a double-stranded protofibril by the formation of the other knob-hole bonds and D-D bonds. Hinge bending as observed in the present simulations can provide the necessary flexibility to accommodate new molecules in the growing fiber. In support of this role, Fig.6C of ref. [8] seems to depict single stranded growth events involving bent fibrin molecules as indicated by arrows 5 and 6. More in general, an imperfect straightening of Fg during integration into a double-stranded protofibril would provide a mechanism for the formation of branch points in the fibrin network.

In the present simulations, several parts of the molecule have been omitted because they are unresolved in the crystal structure, and too long (and disordered) to be meaningfully sampled in the course of our simulations. These include the the *β*N stretch (residues *β*1–57) and the *α*C stretch (residues alpha 201–562). Both parts are mostly disordered (apart from a tendency to form a compact conformation in the C terminal part of the *α*C domain) as revealed by several experimental techniques, including H/D exchange [30], X-ray crystallography [5, 10, 11] and NMR [39]. In fibrinogen, the compact domains of the *α*C region from the two protomers are believed to interact with each other and with the fibrinopeptide B (part of the unresolved *β*B stretch) in proximity of the E region of the molecule, while in fibrin the alphaC domain are believed to move away from the E domain and take part in inter molecular interactions [40]. Although none of these unresolved stretches are known to interact with the coiled coil region of the protein, in principle they could interfere with the bending movements observed in our simulations. The lack of evidence for interactions with the coiled-coil region and the flexible nature of these stretches suggest that their presence will not directly contribute to a stiffening of the hinges of the coiled-coil region. On the other hand, we cannot exclude that their presence may bias bending along particular directions by hindering hinge movements due to excluded-volume effects.

### Flexibility of Fg adsorbed on material surfaces

Fg adsorbed on surfaces often forms tri-nodular structures, as reported by AFM experiments [7, 8]. The three nodules correspond to the E and the two globular D regions of the molecule. The distribution of the *α* angle formed by the three nodules can be used to quantify the flexibility of Fg [7, 8]. The model for Fg emerging from our simulations shows that the flexibility is provided by the presence of the two hinges, while the rest of the molecule does not undergo large conformational changes. Assuming that adsorption does not significantly change this picture, the model can be used to fit the conformational distributions from adsorption data and test various hypothesis on the behavior of Fg. In particular we want to test whether adsorption induces correlations in the behavior of the molecule at the two hinges, which could not be observed in the solution simulations. Such correlations could then be related to specific interactions between the protein and the surface. To this end, we proposed two different models for data fitting: on one hand we used a model where the conformation of one hinge does not affect the conformation of the other, so that the resulting distribution of the experimentally observed *α* angle, because of the symmetry of Fg, can be written as:

$$P\left(\alpha \right)=\int w(\gamma_{1},\varphi_{1})\;w\;(\gamma_{2},\varphi_{2})\;P_{\gamma_{1},\varphi_{1},\gamma_{2},\varphi_{2}}(\alpha )\;d\gamma_{1}d\varphi_{1}d\gamma_{2}d\varphi_{2}$$ (1)

where *γ*_1∣2_ and *φ*_1∣2_ are the bending and torsion angles at the two hinges, respectively (see Methods section), $P_{\gamma_{1},\varphi_{1},\gamma_{2},\varphi_{2}}$(*α*) is the distribution of the *α* angle of adsorbed Fg molecules with given hinge angles *γ*_1_, *φ*_1_, *γ*_2_, *φ*_2_, and *w*(*γ*, *φ*) is the statistical weight of the hinge angle pairs. On the other hand, we proposed a more general model which includes possible correlations between the two hinges:

$$P\left(\alpha \right)=\int W(\gamma_{1},\varphi_{1},\gamma_{2},\varphi_{2})\;P_{\gamma_{1},\varphi_{1},\gamma_{2},\varphi_{2}}(\alpha )\;d\gamma_{1}d\varphi_{1}d\gamma_{2}d\varphi_{2}$$ (2)

where *W*(*γ*_1_, *φ*_1_, *γ*_2_, *φ*_2_) is the combined statistical weight of the conformations of the two hinges. The distributions $P_{\gamma_{1},\varphi_{1},\gamma_{2},\varphi_{2}}$(*α*) were determined using a simple Monte Carlo model (MC) for Fg adsorption (see Methods). The statistical weights *w*(*γ*, *φ*) and *W*(*γ*_1_, *φ*_1_, *γ*_2_, *φ*_2_) for the two models need to be determined by fitting the experimental data. For simplicity, we transformed the integral in Eqs (1) and (2) in a sum over discrete bins defined in the *γ* − *φ* space, so that the weight functions *w*(*γ*, *φ*) and *W*(*γ*_1_, *φ*_1_, *γ*_2_, *φ*_2_) become discrete arrays where each element is the statistical weight of the corresponding bin. The fit is done using a maximum entropy approach, where the statistical weights from our simulations (Fig 2*(e)*) are used as prior knowledge (see Methods for details).

**Fig 4:**
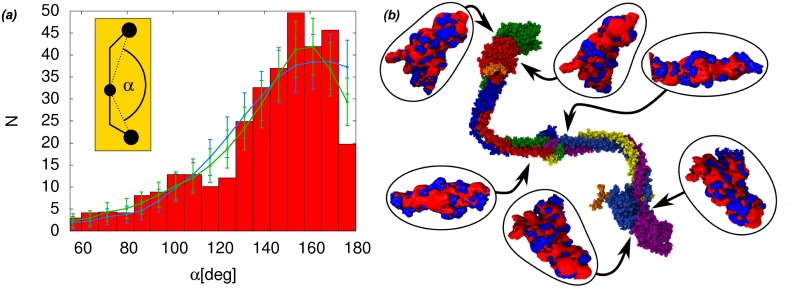
Modelling of fibrinogen adsorption on mica and electrostatic analysis. *(a)* The independent hinge model (blue line, *χ* ^2^ = 1.55) and the general model (green line, *χ* ^2^ = 1.0) for Fg flexibility upon adsorption are fitted to the experimental data (red histograms) of the distribution of the *α* angle in the tri-nodular structures observed by AFM measurements of Fg adsorbed on mica [7]. The error bars on the fit are equal to the square root of the expected count number N in each bin (i.e. assuming a poissonian distribution in each bin). The inset shows the definition of *α* on the simplified Fg model. *(b)* Typical bent conformation of the fibrinogen dimer with the electrostatic potential at D- and E-regions, highlighting the presence of a large negatively charged patch on one side of the surface of the D region. Drawn are the isosurfaces at ±26.7mV (blue/red).

Both models have been used to fit the conformational distribution of Fg adsorbed on mica as observed in AFM experiments [7]. With the independent hinge model, the *χ* ^2^ of the fit remains above the threshold of 5% confidence level for the given number of degrees of freedom, indicating a poor fit. On the other hand, the general model fits the data very well (see Fig 4*(a)*). More generally, it is possible to show that the decoupled weight *w*(*γ*_1_, *φ*_1_)*w*(*γ*_2_, *φ*_2_) of the independent hinge model (Eq 1) would give rise to *P*(*α*) distributions peaked at 180° for any weight function *w*(*γ*, *φ*), which is incompatible with the experimental evidence of a deep trough at 180° [7](Fig 4*(a)*). To see this, we can initially fix the *φ* angles of the two hinges to the same value so that the E and D domains are on the same plane and we can treat the problem as two-dimensional and get a simple analytical expression of *α* as function of *γ*_1_ and *γ*_2_. Then, we can focus on hinges with the same *γ* angle distribution *w*(*γ*, *φ*_0_). A single maximum in this distribution will clearly lead to conformations where both hinges sample the same *γ* angle, which, thanks to the symmetry of the molecule, results in an *α* angle of 180°. But also distributions *w*(*γ*, *φ*_0_) with two (or more) equal maxima, will still lead to *α*-angle distributions with an absolute maximum at 180°, because of the contributions coming from the conformations where the two *γ* angles sample the same maximum and because the peak at 180°, which has only one tail due to being at the end of the definition interval of the *α* angle, will grow twice as big as the other peaks due to symmetrical contributions cumulating on the single tail of the peak (see S3 Fig in Supplementary Information, for an illustrative example). Releasing the constraint on *φ* will not change the situation. The slight drop on the expected counts of the independent hinge model fit at 180° in Fig 4*(a)* is within the numerical uncertainties introduced by binning the MC data.

An analysis of the fitted parameters of the general model, reveals that the largest contributions to the tail of the distribution (95° < *α* < = 110°) corresponding to the lower gaussian peak identified in ref. [7]) come from hinge angle pairs with 60° < *γ*_1_ ≤ 100° and 100° < *γ*_2_ ≤ 140°, that is from fg molecules with one strongly bent and one moderately bent hinge. On the other hand the largest contributions to the 160° peak come from hinge angle pairs with 100° < *γ*_1_ ≤ 140° and 140° < *γ*_2_ ≤ 180°, that is fg conformations with one moderately bent and one almost unbent hinge.

The failure of the independent hinge model and the success of the general model to fit the observed conformational distribution of Fg adsorbed on mica indicates that the conformation of Fg at one hinge affects the conformation at the other hinge upon adsorption on mica. The most probable explanation for this effect is that the propensity to form interactions with mica is not distributed uniformly on the Fg surface. The optimization of the contact surface with mica on both symmetric protomers possibly induces hinge correlations. Since the correlation is observed on a mica surface, which is charged, it is reasonable to assume that the difference between the sides of the Fg protomer has an electrostatic origin.

We have verified the presence of asymmetrically distributed charged patches on the Fg surface by calculating the electrostatic potential generated by the molecule along the simulations. As shown in Fig 4*(b)*, the calculations identify a large negatively charged patch per protomer located on one side of the D region but absent on the other side. This asymmetrical distribution supports our hypothesis regarding the origin of correlations between the two Fg hinges. It has previously been noted that such patches should contribute to the Fg-Fg association during fibrin fibril formation [41]. Here we can identify the D region patch as the a-hole binding site for the fibrinogen A-knob described in reference [42]. Evidently, the involvement of this part of the molecule in interactions with the adsorbing surface may have consequences with respect to fibrin formation.

Unlike the data from Fg adsorption on mica, the data from Fg adsorption on highly oriented pyrolytic graphite (HOPG) rendered hydrophilic with an amphiphilic carbohydrate-glycine modifier(GM) [8] can be fitted with both the general and the independent hinge model at a confidence level larger than 5% (see S4 Fig in Supplementary Information). This may be due to the differences in the conformational distribution induced by a different surface (for example, the 180° bin on HOPG is significantly more populated than on mica, while the tail of the distribution at small *α* angles is less populated than on mica) or due to the lower number of bins/histograms used to report the experimental data on HOPG relative to those on mica in ref. [7] and the overall lower number of data binned, both of which provide less stringent constraints to the fit.

Before closing the subsection we would like to note that, the simplified model of Fg flexibility, emerged from the simulations and used in this section to fit experimental data, may help to refine the models used for describing fibrinogen hydrodynamical properties [19], in the context of adsorption on material surfaces and nanoparticles [20–22], as well as fibrin polymerization [23].

### Interplay between dynamics and binding

Fibrinogen has a multitude of binding partners which help the molecule to carry out its functions. In addition to the plasmin cleavage sites on the coiled-coil region, discussed above, the D region of Fg hosts several other functional binding sites. Two important binding sites are the a- and b-holes, on the *γ*C and *β*C subunit of the D region, respectively, which bind the A- and B-knobs exposed by thrombin upon cleavage of the respective fibrino-peptides. As mentioned already, these binding sites are important for fibrin polymerization as they help to create lateral non-covalent connections between fibrinogen molecules. Surface plasmon resonance experiments showed that binding of fibrino-peptide analogs to the b-hole increases the binding affinity between the a-hole and soluble fibrin fragments containing the A-knob [43]. Hydrogen-deuterium exchange experiments comparing wild-type Fg with the B*β*235Pro/Leu mutant, showed that the mutation, not only leads to a local increase of the flexibility at the *β*C-*γ*C interface, but also alters the flexibility of the loops surrounding the a-hole on the *γ*C domain [29]. In addition, it has been suggested that engagement of the B knobs into the b-holes produces a subtle domain rearrangement in the D regions, favoring lateral aggregation of protofibrils [40]. All these information, thus, suggest that allosteric effects can take place in the D region.

**Fig 5:**
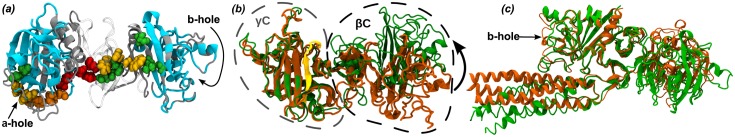
Dynamics in the D region of fibrinogen. *(a)* Visualization of the ensemble of pathways of correlated motion connecting the a- and the b-hole at the two ends of the D region. Pathways connecting all residues in the gray regions have been considered, but only the residues in the blue regions can be actually connected. Residues are colored according to the fraction of pathways they belong to, from red (100%) to green (25%). Thus, all correlated motions occurring at the two ends of the D region involve the residues colored in red. *(b)* Structures of the D region with the largest (orange) and smallest (green) projection along the largest PCA mode. The picture shows the cleft hosting the P1 integrin binding site (in yellow). The two projections can be associated with the open (orange) and closed (green) state of the cleft, and help elucidating how dynamics in the D region may affect binding. *(c)* Structures of the D region and the C-terminal segment of the coiled-coil region (residues *α*125–189, *β*155–458 and *γ*100–394) with the largest and smallest projection along the second largest PCA mode. The picture shows how the opening of the b-hole, indicated by the arrow, is coordinated to the shift of the coiled-coil region away from the *β*C domain.

Our simulations, analyzed using principal component analysis restricted separately to the two globular subunits (*γ*C and *β*C), show that the loops surrounding the a- and b-hole (residues *γ*354–363, *γ*293–302 and *β*382–393, *β*422–432) [44] undergo large correlated fluctuations, as demonstrated by the large weight of those loops in the largest modes of the PCA. To better clarify this aspect, we verified that the dynamics of the a- and b-holes are correlated by the presence of a high correlation pathway (measured using the linear mutual information (LMI) with *r*_*crit*_ ≤ 0.85, see Methods section) connecting them. At the same critical LMI level, several other distant pairs of residues can be connected on the D region including parts of the P1 [45] and P2 [46] integrin binding sites on the *γ*C domain. All the identified pathways pass through the same bottleneck of residues at B*β*204Pro, *γ*216Gly, *γ*217His, *γ*225Glu. More than 75% of all pathways pass through the residues *γ*200Gly, *γ*253Trp and *γ*348Tyr. The identified narrow pathway is shown in Fig 5*(a)*. Some of these residues are in contact with the residues locally perturbed upon the B*β*235Pro/Leu mutation, which may explain why the effects of this mutation reach the a-hole. With the exception of *γ*217His all residues belonging to the bottleneck of the high correlation pathways are absolutely conserved among vertebrates (sequences discussed above). In addition, the same residues appear to be associated with disease-inducing mutations in humans [47]. These data, taken together, reinforce the hypothesis of an allosteric network connecting the a- and b-holes.

The integrin binding sites represent another important element of the functionalities of Fg, involved in facilitating immune response. Several residues of the *γ*C domain have been implicated in integrin binding [45, 46]. Our simulations show that the accessibility of the P1 region of the integrin binding site is affected by the large scale fluctuations of the D region and of the loops around it. Indeed the three largest PCA modes of the D region involve rigid rotations of the *β*C relative to *γ*C domains, which directly alter the P1 accessibility. In addition, these modes have large components on two loops surrounding the P1 binding site, *γ*354–363 and *β*280–285, whose coordinated fluctuations significantly affect P1 accessibility. The opening/closing mechanism of the P1 cleft described by the largest of the PCA modes is characterized by a change in distance between the integrin binding site P1 and the *β*C loop B280-B285 from about 1.2nm to 1.9nm (Fig 5*(b)*).

Extending the PCA analysis to include also the C-terminal segment of the coiled-coil region (residues *α*125–189, *β*155–458 and *γ*100–394) reveals that the relative rotations of the *β*C and *γ*C domains described above are associated with movements of the C-terminal segment of the coiled-coil region relative to the D domain. In particular, the second largest PCA mode describes a motion where the distance between the *β*C domain and the coiled-coil region increases in combination with an opening of the b-hole (Fig 5*(c)*). This mechanism recalls very closely the one which has been hypothesized to explain how the interaction between B-knob and b-hole induces a slight conformational change which favors the lateral aggregation of protofibrils [40].

### Conclusion

The classical atomistic molecular dynamics simulations of fibrinogen in solution reveal the extraordinary flexibility of the molecule resulting in large bending motions of the coiled-coil regions, favored by the presence of two hinges. The hinges are linked to a non-helical segment in the coiled-coil region of the *γ* chain, a feature conserved across vertebrates. This may indicate a possible functional role for the bending motions. In the simulations, the bending of the coiled-coil region helps to expose the early plasmin cleavage sites. A simplified model of Fg flexibility has been derived from the simulations and used to test hypothesis about Fg adsorption by fitting AFM data. This lead to hypothesize correlations between the two hinges upon adsorption on mica. A probable cause for the correlations is an asymmetric distribution of charged patches on the surface of the molecule and in particular on the D globular regions, which is observed in the simulations. We anticipate that the simplified model presented here could lead to more accurate estimates of the hydrodynamic properties of Fg. Furthermore, an analysis of the pathways joining residues with highly correlated motions in the simulations hints at an allosteric regulation of the binding at the a- and b-hole in the D region of Fg.

## Methods

### Atomistic Molecular Dynamics simulations

The simulations are based on the crystal structure of human Fg (PDB ID: 3GHG) [5]. The carbohydrate groups that are only partly resolved in the crystal have been modelled using VMD and introduced in some of the simulations. The unresolved parts of the protein structure (the *α*C domain and the N terminal segments of all the chains) have not been included in the calculations. Several molecular constructs have been prepared to assess the role of the different components of the Fg molecule. The effects of the carbohydrate chains on the dynamics of Fg have been investigated by simulating both the unglycosylated system, the sytem glycosylated at residue *β*364 (mono-glycosylated) and the system glycosylated at residue *β*364 and *γ*52 (di-glycosylated). Protomer-protomer interactions were investigated by simulating both the full fibrinogen dimer (6 protein chains) and the protomer system (3 chains). Although the single or isolated protomer has never been observed experimentally, the reason for studying it are the following: 1) the two protomers in the dimer are identical, i.e. they will show a similar behavior, 2) since we neglect the unresolved parts of the molecule, the two protomers interact only through the small dimerization interface, i.e. their reciprocal influence is limited, 3) A ring of disulphide bridges covalently bonding the three chains in the N-terminal part of the coiled-coil region dramatically reduces the influences of the dynamics of the dimerization domain on the rest of the molecule (see S1 Fig in Supplementary Information). In addition to that, the simulations of the protomer take much less computational time than the dimer i.e. they can be extended to significantly larger time scales.

Rectangular simulation boxes with explicit TIP3P water [48] and physiological ion concentration (150 mMol [NaCl]) were prepared using VMD [49] (Table 1 for box sizes).

**Table 1:** List of the performed simulations.

System	Initial box size [nm]	N^a^	Simulation time^b^ [ns]
**Dimer**			
mono-glycosilated	13.27 × 48.59 × 12.70	788173	77, 88
unglycosilated	13.27 × 48.59 × 12.70	786811	25, 20
**Protomer**			
di-glycosilated	11.02 × 35.07 × 10.42	381304	45, 21, 21
mono-glycosilated	12.28 × 27.89 × 11.57	381397	199, 188
unglycosilated	12.28 × 27.89 × 11.57	380169	135, 109, 100, 82, 51, 43, 30, 20, 20, 14

^a^ Total number of particles in the system.

^b^ Each number indicates the time length of an independent simulation.

Isobaric-isothermal simulations were set up at a temperature of 310K and pressure of 1atm using NAMD [50] with a Langevin thermostat and a Langevin piston barostat [51, 52] using 200 ps^−1^ and 100 ps^−1^ as decay time, respectively. The covalent bonds involving hydrogen atoms were fixed in length and a 2fs timestep was used. The CHARMM22 force field with CMAP corrections [53] was used with its recent extension to carbohydrates [54] in combination with ParamChem (http:/www.paramchem.org) and the CHARMM generalized force field (CGenFF) [55]. This force field has been already tested in a large variety of systems and found very reliable in reproducing several biophysical properties, including also the folding process of proteins [56] where it was shown to provide results very similar to other popular force fields (several versions of AMBER and modifications of CHARMM) in the characterization of the native state of proteins. The van der Waals forces were cut off at 1.2nm while PME was used for long range electrostatic interactions with a grid spacing of 1Å. After energy minimization (NAMD’s conjugate gradient algorithm, 15000 steps) of hydrogen atoms and water molecules, the system was heated and equilibrated for 10ns. Production runs statistics are given in Table 1. We employed collective variable constraints (distanceXY, directiondir or orient) to keep the main axis of the molecule aligned to the simulation box and verified that this had no influence on the overall dynamics by comparing to unconstrained simulations.

To identify the collective motions of the whole Fg molecule and of its subdomains we performed several principal component analyses (PCA) [57] using wordom [58] and GROMACS utilities [59]. DynDom [28] was used to identify rigid domains and hinges of motion. The overlap between spaces spanned by the dominant PCA modes of different simulations was used to quantify the similarity of the observed dynamics [60]. The overlap is defined as:

$$O\left(\left\{{\underline{x}}_{i}\right\},\left\{{\underline{y}}_{i}\right\}\right)=\frac{1}{n}\sum_{i=1}^{n}\sum_{j=1}^{n}{\left({\underline{x}}_{i}\cdot {\underline{y}}_{j}\right)}^{2}\;,$$ (3)

where $\left\{{\underline{x}}_{i}\right\}$ and $\left\{{\underline{y}}_{j}\right\}$ are the two subspaces spanned by the principle components ${\underline{x}}_{1}\ldots {\underline{x}}_{n}$ and ${\underline{y}}_{1}\ldots {\underline{y}}_{n}$, respectively.

The linear correlation coefficient between two variables is defined as the ratio between the covariance of the two variables and the product of the two standard deviations, $r=\sum \left(x_{i}-\bar{x})(y_{i}-\bar{y}\right)/\sqrt{\sum {\left(x_{i}-\bar{x}\right)}^{2}{\left(y_{i}-\bar{y}\right)}^{2}}$. If *P*_1_(*t*), *P*_2_(*t*), *P*_*N*_(*t*) are the projections of the trajectory at time *t* along the 1st, 2nd and Nth PCA components, respectively, a linear combination *CP*(*t*) of PCA projections is obtained as *CP*(*t*) = ∑_*j*_ *a*_*j*_ *P*_*j*_(*t*), where *a*_*j*_ are the coefficients of the combination. As mentioned in the Results section, a very large correlation coefficient (0.96) between *CP*(*t*) and the *γ* angle was obtained by using *a*_1_ = 1, *a*_2_ = −1 and *a*_3_ = −0.1 (and all the other *a*_*j*_ = 0).

Apart from PCA, as a further measure of correlation between the movement of the residues, the linear mutual information (LMI) [61] was used which is defined as follows:

$$LMI\left(x_{i},x_{j}\right)=\frac{1}{2}(\ln[\det C_{i}]+\ln[\det C_{j}]-\ln[\det C_{ij}])$$ (4)

where ***x***_*i*_ is the position of the *i*th C_*α*_ atom, $C_{i}=\left\langle x_{i}^{T}\;x_{i}\right\rangle$ and *C*_*ij*_ = ⟨(*x*_*i*_ − ⟨*x*_*i*_⟩)(*x*_*j*_ − ⟨*x*_*j*_⟩)⟩

The LMI matrix was calculated for mono-glycosilated fibrinogen simulations. Based on it, a network was built with each node representing a residue. Nodes are connected if their LMI exceeds a threshold *r*_*crit*_. This parameter was successively reduced until a pathway between the a- and b-holes was identified at *r*_*crit*_ = 0.85. The shortest path connecting the two binding pockets is determined in this network. To save computation time only pathways of a maximum length *N* where considered such that $r_{crit}^{N}>0.025$. As a control, pathways between all residues that are at least 6nm apart were identified.

The electrostatic potential of the globular domains of fibrinogen was calculated by solving the Poisson-Boltzmann equation with the APBS [62] software over a series of similar aligned structures. Then, the electrostatic potential was averaged over the structures. The time averaged potential for each domain was calculated by averaging the potential at each grid point over all snapshots.

### Sequence comparison

Sequences of Fg were identified by searching the UniProt data base [63]. In this search, sequences of 83 different species were identified that contained the *γ* chain at least up to the hinge region. Complete sequences of all three chains in the coiled-coil region where found for 33 species. Sequence alignments were performed using Chimera [64], and the analysis of structural disorder was performed using the program DisEMBL [33].

### Simplified model

**Fig 6:**
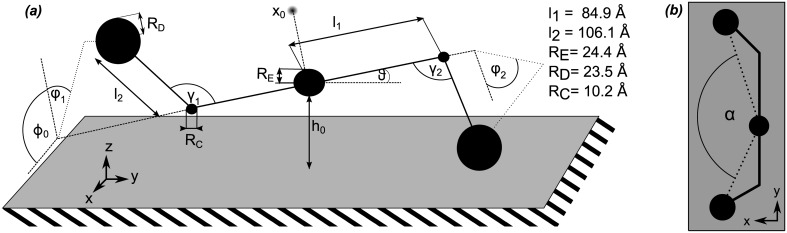
A simplified model of fibrinogen adsorption. *(a)* Simplified model of fibrinogen flexibility. The hinge bending angles (*γ*_1_, *φ*_1_, *γ*_2_ and *φ*_2_) represent the only degrees of freedom of the molecule in this representation. The geometrical parameters are determined from the crystal structure of Fg. *(b)* 2D projection of the model as seen in AFM experiments.

A simplified representation of Fg adsorption has been developed to map Fg conformations represented by the hinge angles to the conformations observed in AFM experiments, characterized by the *α* angle between the globular domains (see Fig 6). The model for Fg is built around a central rod, representing the stiff coiled-coil regions, which connects the two hinges. The E region is represented as a sphere placed at the center of the rod. The D regions are also represented as spheres connected to the hinges with rods, that can pivot around the hinge. The dimensions of the model components are extracted from the crystal structure (Fig 6). An additional point (*x*_0_ in Fig 6) on the surface of the E region is used as a reference for the measure of the torsion angles at the two hinges. Within this simplified representation, adsorption occurs if and only if the distance of the E and the two D regions of a Fg molecule from the adsorbing surface (represented by an ideal plane) is lower than a threshold *h*_max_ and both hinges lie above the surface.

We use a simple Monte Carlo (MC) algorithm to generate a large set (4 ⋅ 10^7^) of coarse grained adsorbing Fg conformations. The MC algorithm consists of creating adsorbed conformations by randomly drawing hinge angles for one protomer and placing it such that the first D region as well as the E region contact the surface. The bending angle *γ* for the second hinge is randomly drawn as well, while the value of *φ* for the second hinge is calculated to ensure that the second D-region is in contact with the surface. The resulting conformations are accepted in such a way that a uniform distribution of *γ* and *φ* is obtained at both hinges. For each accepted conformation we measure the hinge angles, as well as the *α* angle (Fig 6) between the globular regions, as it would be observed in an AFM experiment, i.e., we project the regions’ centers on the surface and measure the angle between the three points.

To fit the experimentally observed distribution of the *α* angle we divided the *γ* − *φ*-plane characterizing the conformation of one hinge into 12 rectangular bins, indexed accordingly. Each adsorbing Fg conformation can be characterized by the two discrete indexes *i* and *j* of the (*γ*, *φ*) region where its two hinges occur. We then determine the bending distribution *P*_*ij*_(*α*) observed in the conformations sampled with MC from each pair of bins *i* and *j* of the (*γ*–*φ*) plane (see S2 Fig in the supporting information). It is important to note that, not all *ij* pairs lead to adsorbing structures. We exclude the non-adsorbing *ij* pairs (bins) from the computation. We then assume that the observed experimental distribution *P*(*α*) is a superposition of the distributions from the adsorbing conformations of Fg in each pair of bins. The most general form of superposition is given by *P*(*α*) = ∑_*ij*_ *a*_*ij*_ *P*_*ij*_(*α*), which is the discretized version of Eq 2, where the parameters *a*_*ij*_ replace the continuous weights *W*(*γ*_1_, *φ*_1_, *γ*_2_, *φ*_2_). The *a*_*ij*_ parameters can then be fitted to the experimental distribution. If the two hinges behave independently, then, given the symmetry of the molecule, an independent hinge model *P*(*α*) = ∑_*ij*_ *a*_*i*_ *a*_*j*_ *P*_*ij*_(*α*) should be able to fit the data. This model is the discretized version of Eq 1, where the parameters *a*_*i*_ replace the continuous weights *w*(*γ*, *φ*). In this case, we will have only the *a*_*i*_ parameters, i.e., one parameter for each bin in *γ* − *φ*-space.

Both the general and the independent hinge model are fitted to the experimental data using the maximum-entropy method [65], where the Shannon entropy of the parameters is maximized with respect to the *a priori* knowledge of the system, with the constraint that the reduced *χ* ^2^ = ∑_*α*_(*N*(*α*) − *N*_*tot*_ *P*(*α*))^2^/*N*_*tot*_ *P*(*α*) equals 1, where *N*(*α*) are the histograms of the *α* angle from the experiments, and *N*_*tot*_ is the sum of the histograms. The expression that is maximized for the general model is:

$$J=-\sum_{ij}a_{ij}\log\left(a_{ij}/m_{ij}\right)-\lambda \;(\chi^{2}-1)-\alpha \;(F-1)$$ (5)

where *m*_*ij*_ is the bias distribution for the parameters, *F* is the normalization function of the parameters *F* = ∑_*ij*_ *a*_*ij*_ and *λ* and *α* are Lagrange multipliers to impose the constraints on *χ* ^2^ and normalization. A similar expression is used for the independent hinge model with the necessary modifications. The bias distribution *m*_*ij*_ represents the information already known about the parameters before fitting the data. The statistical weight *w*_*i*_ of each *γφ* bin as measured in the atomistic simulations (Fig 2*(e)*) were used to estimate the *m*_*ij*_ = *w*_*i*_ *w*_*j*_.

## Supporting Information

S1 Movie Movie showing the bending motion occurring at the hinge on the coiled-coil region of fibrinogen from a selected molecular dynamics trajectory. (MP4)

S1 Fig Root mean square fluctuations of the C_*α*_, N and C backbone atoms of the N terminal residues of Fg (up to the middle of the coiled-coil region, 10 residues before the identified hinge) for the protomer (red) and dimer (blue) simulations. The *α*, *β* and *γ* chain are reported in *(a)*, *(b)* and *(c)*, respectively. The vertical black line indicates the location of the ring of disulphide bridges at the N-terminus of the coiled-coil region. The larger fluctations in the N-terminal part of the protomer, due to the absence of stabilizing interactions with the other protomer, are localized to the very first residues of the chain and do not affect the dynamics of the rest of the molecule. (PDF)

S2 Fig The distributions of the *α* angle (*P*_ij_(*α*)) from selected regions of the *γ*–*φ* space. (PDF)

S3 Fig Relation between the hinge angles and the *α* angle in the independent hinge model. At a fixed *φ*_1_ = *φ*_2_ any distribution of the two *γ* angles (example of a double peak, left panel), gives rise to an *α* angle distribution strongly peaked at *π* (i.e., 180°, right panel). This is incompatible with the AFM data of Fg adsorption on mica [7] (Fig 4*(a)*), which show that the 180° region is poorly populated. (PDF)

S4 Fig The general (green line) and independent hinge (blue line) models of Fg fit the data of Fg adsorption on HOPG from Ref. [8] both with a *χ* ^2^ of 1.0. The errors on the fit are equal to the square root of the expected count number N in each bin (i.e. assuming a poissonian distribution of N in each bin). The good fit of the independent hinge model, in this case, may be determined either by the fact that the experimental distribution shows a less deep trough at 180° and a less populated tail at low *α* angles than the one obtained for Fg adsorption on mica [7], or by the less stringent contraints on the fit due to the lower number of bins used to represent the experimental data in ref. [8] and the lower total number of binned data than those provided for the Fg adsorption on mica in ref. [7]. (PDF)
