# Supplementary figures and images for: The Internal Dynamics of Fibrinogen and Its Implications for Coagulation and Adsorption
Source: PLoS Comput Biol. 2015 Sep 14;11(9):e1004346. doi: 10.1371/journal.pcbi.1004346 (PMC4569070; doi:10.1371/journal.pcbi.1004346)

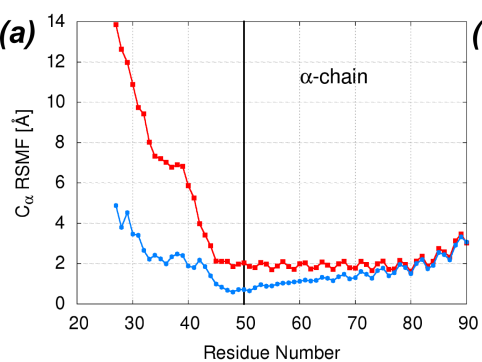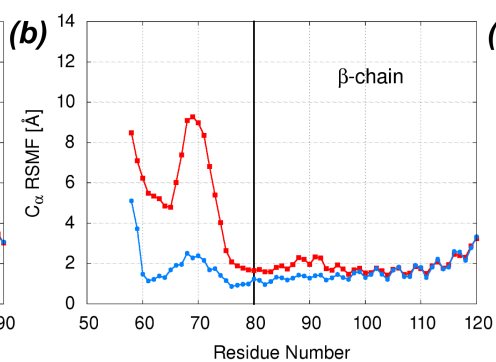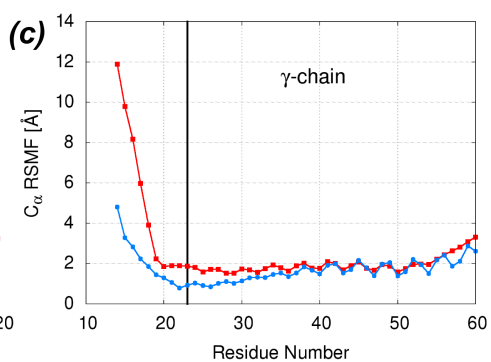

Supplement: S1 Fig — (PDF) [file pcbi.1004346.s002.pdf]

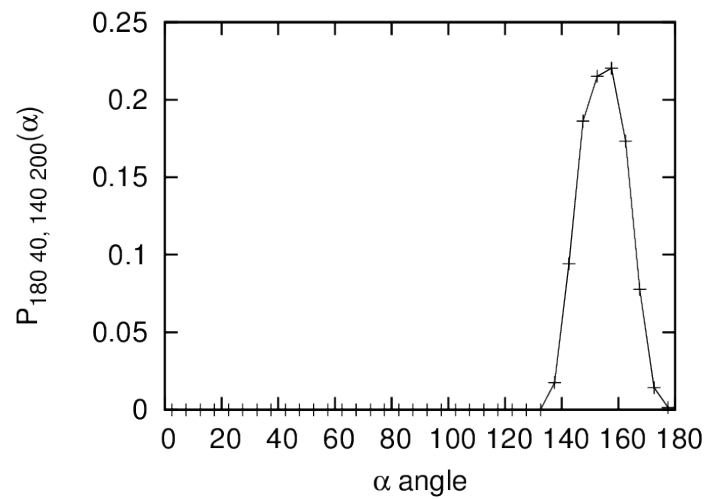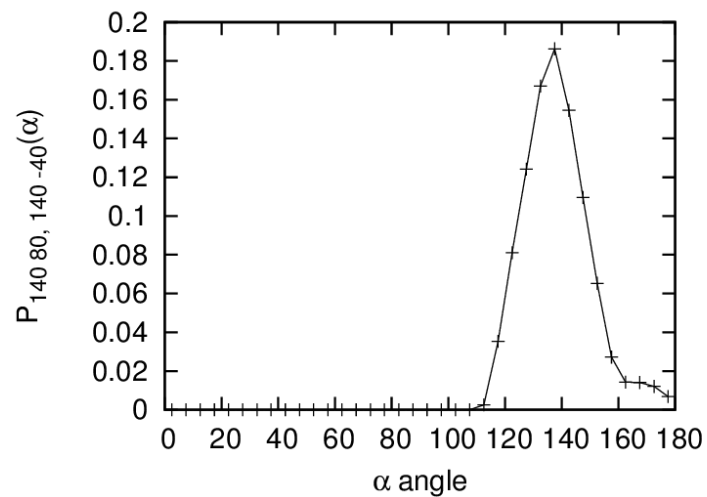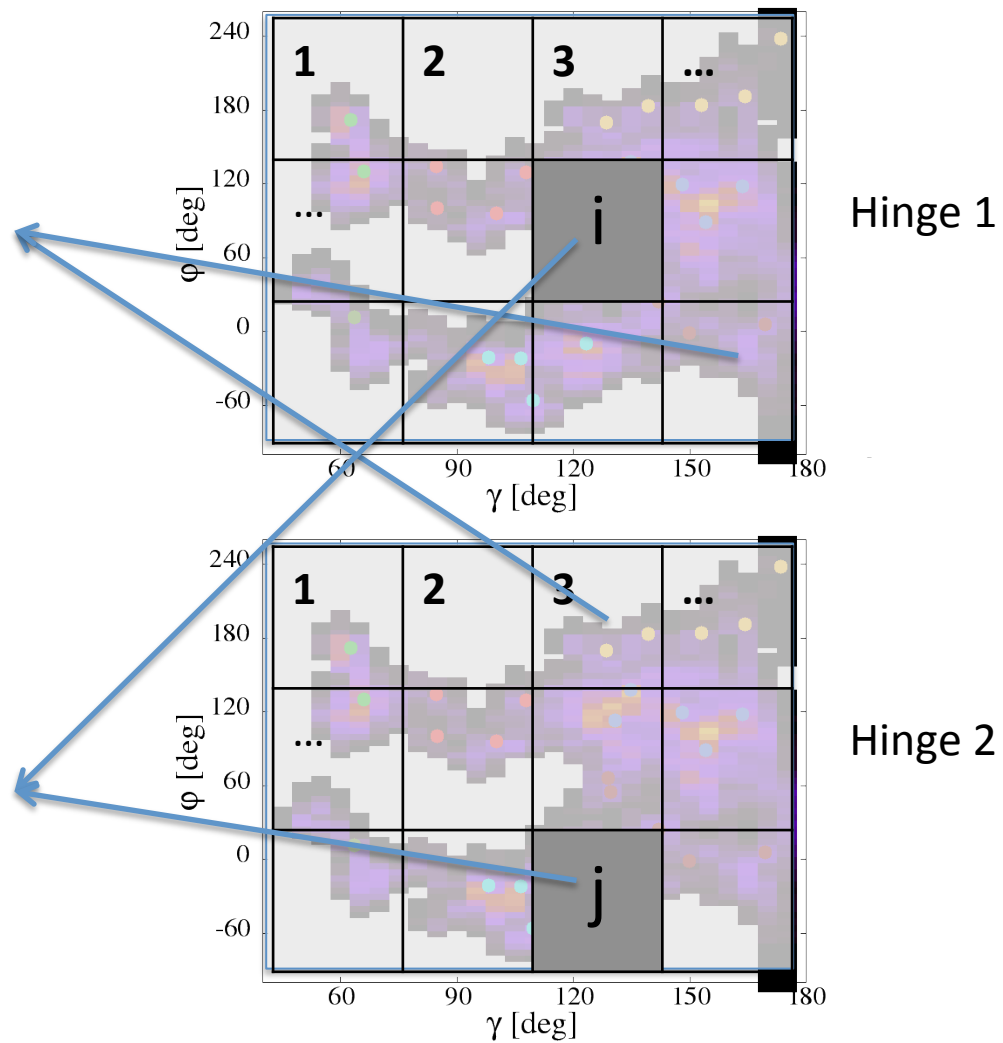

Supplement: S2 Fig — (PDF) [file pcbi.1004346.s003.pdf]

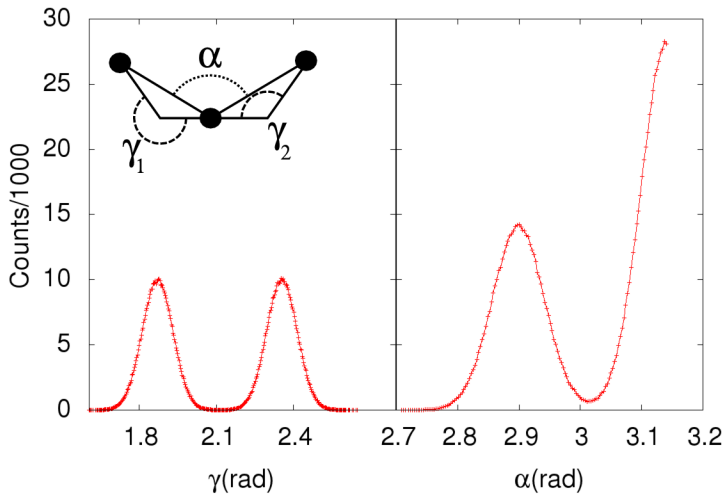

Supplement: S3 Fig — (PDF) [file pcbi.1004346.s004.pdf]

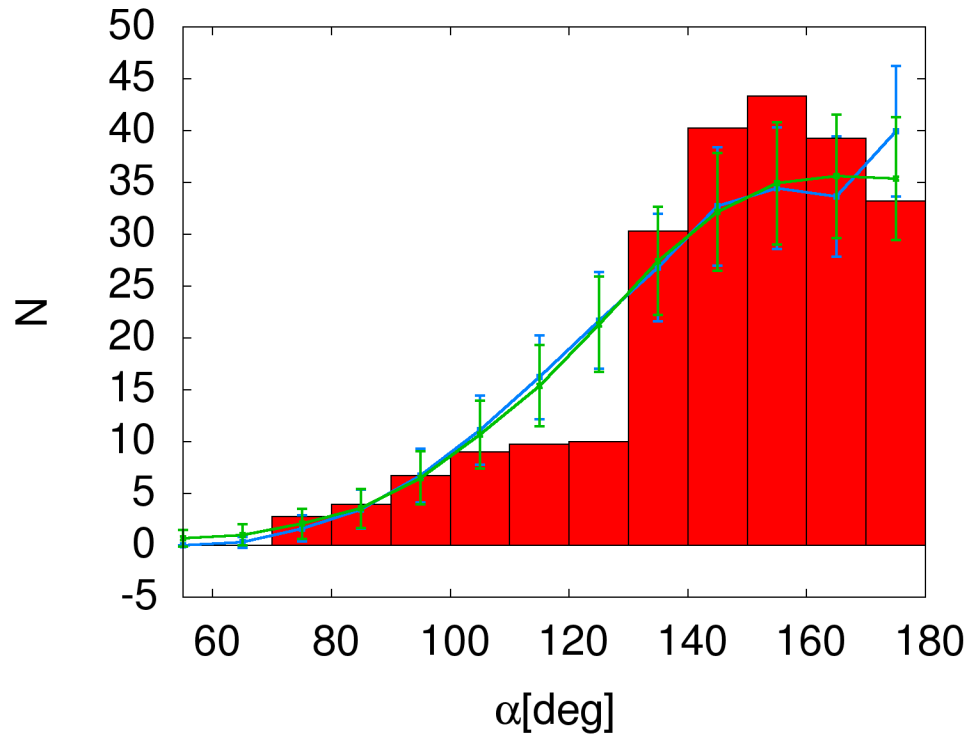

Supplement: S4 Fig — (PDF) [file pcbi.1004346.s005.pdf]
